# Supplementary material for: Implants for HIV prevention in young women: Provider perceptions and lessons learned from contraceptive implant provision
Source: PLoS One. 2022 Jan 13;17(1):e0262043. doi: 10.1371/journal.pone.0262043 (PMC8758078; doi:10.1371/journal.pone.0262043)
Supplement: S1 Appendix — (PDF) [file pone.0262043.s001.pdf]

Staff Administered

Topic Guide for In-depth interviews:

Group 5 - Nurses:

Hello, we are so happy that you agreed to speak with us today, we hope that you will feel free to talk with us. Please remember to tell me if you would like to take a break during the interview.

Outline house rules: confidentiality of information; underscore no right or wrong answers.

As you may be aware even though women bear a disproportionate burden of HIV infection in sub-Saharan Africa there are limited options available to them to prevent HIV infection. A new method is the use of anti-retrovirals in the form of daily tablets of a combination of tenofovir + emtricitabine. To work, these tablets have to be taken every day and this is a challenge for some women. Scientists in different parts of the world are developing ways to overcome the adherence challenges through a range of novel approaches. CAPRISA is working on developing an annual antiretroviral sub-dermal implant for HIV prevention in women. This implant is similar to a contraceptive implant. We are undertaking interviews with users and providers to understand their experiences and perceptions of the contraceptive implant so that this information can be used to inform the development and testing of an ARV based implant for HIV prevention in the future. We would like to ask you a few questions about your experiences with contraceptive implant provision and your perceptions about users perspectives

	Demographics	*Answers*
	**Age (years)**	
	**Gender**	☐ *Female* ☐ *Male*
	**Clinic Name:**	
	**Qualification:**	
	**Approximate years of experience with Implanon NXT®**	
	**Number of Implanon NXT® insertions performed in the last year?**	☐ *0* ☐ *1-10* ☐ *10-100* ☐ *>100*
	**Number of Implanon NXT ®removals done in the last year?**	☐ *0* ☐ *1-10* ☐ *10-100* ☐ *>100*
	**Reasons for Implanon NXT® removal (ever removed, tick all that apply)**	☐ *Indication period finished (end of device lifetime i.e. 3 years)*<br>☐ *No longer wants contraception*<br>☐ *Irregular menstrual bleeding*<br>☐ *Insertion complications (e.g. Infection, expulsion)*<br>☐ *Pregnant while on implant*<br>☐ *other: specify:*<br>______________________________<br>______________________________

Interviewer Initials: ☐☐☐ **Date:** ☐☐/☐☐☐/☐☐

	Training and knowledge of Implanon NXT®	
	Have you undergone any training regarding Implanon NXT® insertion?	☐ *Yes* ☐ *No*
	If yes, what training did you undergo with regards to the insertion of the Implanon NXT®?	☐ *Not applicable*<br>*Answer:*
	Why or how were you chosen to for this training?	
	Were you trained on how to remove Implanon NXT®?	☐ *Yes* ☐ *No*
	What was covered during the training?<br>Theoretical component?<br>Practical component?	
	How many days was the training?	
	Who conducted the training?	
	Out of the scale of 5, where 5 is the best and 0 is the poorest, how would you rate this training?	☐ *0* ☐ *1* ☐ *2* ☐ *3* ☐ *4* ☐ *5*
	How many people were trained from your clinic?	
	What was missing during the training?	
	What was unnecessary in the training?	
	What mentorship was offered after the training?<br>Who offered the mentorship and how?<br><br>How was this helpful to you?	
	In your contraceptive counselling, do you explain to women the mode of action for Implanon NXT® in preventing pregnancy?<br>If yes, what were the key things that you told them?	☐ *Yes* ☐ *No*<br><br>☐ *Not applicable*<br>*Answer:*
	Do you give any special instructions coupled with this insertion?<br>What were those instructions?	☐ *Yes* ☐ *No*<br><br>☐ *Not applicable*<br>*Answer:*

Interviewer Initials: ☐☐☐ **Date:** ☐☐/☐☐☐☐/☐☐

Attitudes and perceptions of Implanon NXT®		
	According to you, as a healthcare professional:	
	What is the most popular contraceptive method amongst users / patients? Why?<br>What is your favourite? Why?	
	What are the benefits of the implant?	
	What are the disadvantages?	
	What do you personally like about the implant or Implanon NXT®?	
	What do you personally not like about the implant Implanon NXT®?	
	What do you think interested women in this method?<br>What do you think they considered to be the benefits for them	
	What do you think, made women not interested?<br>Many women asked for the premature removal of the contraceptive implant. Why do think this happened?	
	What did the women say is the reason to have the contraceptive implant removed prematurely?	
Closing comments on Implanon NXT®		
	Do you have any special case that is worth mentioning?	

Interviewer Initials: ☐☐☐ **Date:** ☐☐/☐☐☐/☐☐

**_Implants for HIV-prevention (based on your experience of the contraceptive implant roll-out)_**<br>Current options for preventing HIV infection may not suit everyone. Not everyone is able to use condoms consistently with every sex act. Oral PrEP (anti-retrovirals in the form of daily tablets of a combination of tenofovir + emtricitabine) has to be taken every day and this is a challenge for some people. Scientists in different parts of the world are developing ways to overcome the adherence challenges through a range of new approaches. CAPRISA is working on developing an implant for HIV prevention. This implant is similar to a contraceptive implant, but does not contain the drug to prevent pregnancy, it contains an antiretroviral to prevent HIV-infection, so side effects may be different. It may also not be able to offer protection for 3 years, perhaps only 1 year. It may also mean having to insert at least two implant rods at a time.		
	As a Healthcare Professional, what characteristics would you prefer in an implant for HIV-prevention?<br>*What should the rods be like?*<br>*SIZE:*<br>• *Larger rod but used for longer? Smaller rod but only effective for shorter amount of time?*<br>*DURATION:*<br>• *What time period should the user be able to use a rod for? (probe for minimum time)*<br>*PALPABILITY:*<br>• *Do you think it should be felt?*<br>• *Explore whether they think it should be stiff/flexible*<br>*Where should the implant be placed in the body?*<br>• *Explore what they think would be acceptable and what would not be.*<br>*Ask whether the implant should be biodegradable or if it should be removed at a clinic.*	
	What do you think the community / patients would look for in an implant for HIV-prevention?	
	What should researchers bear in mind when considering an implant for HIV-prevention?	

Interviewer Initials: ☐☐☐ **Date:** ☐☐/☐☐☐☐/☐☐

Community Engagement		
	Was the community ever engaged regarding the introduction of the contraceptive implant Implanon NXT®?	☐ *Yes* ☐ *No*<br>☐ *Not aware of any community engagement* ***(SKIP to Closing Comments)***
	At what stage was this done?	
	Who was targeted in the community during this engagement?	
	What was the content of engagement?	
	Discuss the activities that were done to introduce this contraceptive implant Implanon NXT® to young people?	
	Were men involved? If so, how?	☐ *Yes* ☐ *No*<br>*Answer:*
	Who, amongst the staff was responsible for this?	
	What lessons did you learn during the community engagement for the contraceptive implant that would be useful when engaging community about an implant for HIV prevention?	

Interviewer Initials: ☐☐☐ **Date:** ☐☐/☐☐☐/☐☐

Staff Administered:

Topic Guide for In-depth interviews:

Group 5 – Community Healthcare Workers:

Hello, we are so happy that you agreed to speak with us today, we hope that you will feel free to talk with us. Please remember to tell me if you would like to take a break during the interview.

Outline house rules: confidentiality of information; underscore no right or wrong answers.

As you may be aware even though women bear a disproportionate burden of HIV infection in sub-Saharan Africa there are limited options available to them to prevent HIV infection. A new method is the use of anti-retrovirals in the form of daily tablets of a combination of tenofovir + emtricitabine. To work, these tablets have to be taken every day and this is a challenge for some women. Scientists in different parts of the world are developing ways to overcome the adherence challenges through a range of novel approaches. CAPRISA is working on developing an annual antiretroviral sub-dermal implant for HIV prevention in women. This implant is similar to a contraceptive implant. We are undertaking interviews with users and providers to understand their experiences and perceptions of the contraceptive implant so that this information can be used to inform the development and testing of an ARV based implant for HIV prevention in the future. We would like to ask you a few questions about your experiences with contraceptive implant provision and your perceptions about users perspectives

	Demographics	*Answers*
	**Age (years)**	
	**Gender**	☐ *Female* ☐ *Male*
	**Clinic Name:**	
	**Years of service as a CHW**	
	**What has been the "good times" as a CHW?**	
	**What has been the challenges you experienced as a CHW?**	

Interviewer Initials: ☐☐☐ **Date:** ☐☐/☐☐☐☐/☐☐

	Knowledge and experiences of Implanon NXT®	
	What is your role in the contraception service?	
	What is the most popular method amongst users / patients?<br>Why?<br>What is your favourite contraception method?<br>Why?	
	Were you involved with the contraceptive implant?	□ *Yes* □ *No*
	If yes what was your involvement?	
	What went well with this implant?	
	What did not go well?	
	If DOH is to start all over again <u>with introducing a new implant method</u> to communities, what suggestions would you give them?	
	Have you undergone any training on contraceptive services / methods available at the local PHC clinics?	□ *Yes* □ *No*
	What was covered during the training?	
	How many days was the training?	
	Who conducted the training?	
	Out of the scale of 5, where 5 is the best and 0 is the poorest, how would you rate this training?	□ *0* □ *1* □ *2* □ *3* □ *4* □ *5*
	What was missing during the training?	
	What was unnecessary in the training?	
	What mentorship was offered after the training?<br><br>Who offered the mentorship and how?<br><br>How was this helpful to you?	

Interviewer Initials: ☐☐☐ **Date:** ☐☐/☐☐☐/☐☐

Implants for HIV-prevention (based on your experience of the contraceptive implant roll-out)<br>Current options for preventing HIV infection may not suit everyone. Not everyone is able to use condoms consistently with every sex act. Oral PrEP (anti-retrovirals in the form of daily tablets of a combination of tenofovir + emtricitabine) has to be taken every day and this is a challenge for some people. Scientists in different parts of the world are developing ways to overcome the adherence challenges through a range of new approaches. CAPRISA is working on developing an implant for HIV prevention. This implant is similar to a contraceptive implant, but does not contain the drug to prevent pregnancy, it contains an antiretroviral to prevent HIV-infection, so side effects may be different. It may also not be able to offer protection for 3 years, perhaps only 1 year. It may also mean having to insert at least two implant rods at a time.		
	As a Community Healthcare Worker, what characteristics would you prefer in an implant for HIV-prevention?<br>*What should the rods be like?*<br>*SIZE:*<br>• *Larger rod but used for longer? Smaller rod but only effective for shorter amount of time?*<br>*DURATION:*<br>• *What time period should the user be able to use a rod for? (probe for minimum time)*<br>*PALPABILITY:*<br>• *Do you think it should be felt?*<br>• *Explore whether they think it should be stiff/flexible*<br>*Where should the implant be placed in the body?*<br>• *Explore what they think would be acceptable and what would not be.*<br>*Ask whether the implant should be biodegradable or if it should be removed at a clinic.*	
	What do you think the community / patients would look for in an implant for HIV-prevention?	
	What should researchers bear in mind when considering an implant for HIV-prevention?	

Interviewer Initials: ☐☐☐ **Date:** ☐☐/☐☐☐/☐☐

Community Engagement		
	Was the community ever engaged regarding the introduction of the contraceptive implant?	☐ *Yes* ☐ *No*<br>☐ *Not aware of any community engagement* ***(SKIP to Closing Comments)***
	Were you at all involved with the introduction of the contraceptive implant into the community?	☐ *Yes* ☐ *No*
	If yes, what was your role?	
	How was the community informed about the contraceptive implant?	
	Who in the community was informed about the contraceptive implant?	
	Were men involved?<br>If so, how?	☐ *Yes* ☐ *No*<br>*Answer:*
	By whom was the community informed about the contraceptive implant?	
	What was the message that was sent to the community?	
	Discuss any activities that were done to introduce this contraceptive implant method to young people?	
	What lessons did you learn during the community engagement for the contraceptive implant that would be useful when engaging community about an implant for HIV prevention?	

Interviewer Initials: ☐☐☐ **Date:** ☐☐/☐☐☐☐/☐☐
