# Supplementary material for: Implants for HIV prevention in young women: Provider perceptions and lessons learned from contraceptive implant provision
Source: PLoS One. 2022 Jan 13;17(1):e0262043. doi: 10.1371/journal.pone.0262043 (PMC8758078; doi:10.1371/journal.pone.0262043)
Supplement: Attachment — Submitted filename: PONE-D-21-27516-GSBreview-comments.pdf [file pone.0262043.s002.pdf]

PLOS ONE

Implants for HIV prevention in young women: Provider perceptions and lessons learnt from contraceptive implant provision

--Manuscript Draft--

**Manuscript Number:**	PONE-D-21-27516
**Article Type:**	Research Article
**Full Title:**	Implants for HIV prevention in young women: Provider perceptions and lessons learnt from contraceptive implant provision
**Short Title:**	Implants for HIV prevention: Provider perceptions and lessons learnt from contraceptive implant provision
**Corresponding Author:**	Hilton Humphries, MA (Research Psychology)<br>Centre for the Aids Programme of Research in South Africa<br>Durban, KwaZulu-Natal SOUTH AFRICA
**Keywords:**	HIV; Contraceptive implants; HIV prevention; Young women, Health-care workers
**Abstract:**	Preventing new HIV infections, especially amongst young women is key to ending AIDS as a public health threat. HIV prevention implants (PrEP implants) currently in clinical development, offer a new technology to expand the HIV prevention toolbox. Feedback from providers and future users at early clinical development stages is important to identify which potentially modifiable characteristics may improve acceptability and uptake. Healthcare workers (HCWs) play an important role in the provision of new technologies and have been instrumental in the provision of contraceptive implants. HCWs perspectives and lessons learned during the rollout of contraceptive implants will allow us to understand what factors may impact the roll-out of PrEP implants. We conducted 18 interviews with HCWs (9 nurses and 9 community healthcare workers) in rural KwaZulu-Natal, South Africa. HCWs listed the longacting nature of the contraceptive implant as a key benefit, helping to overcome healthcare system barriers like heavy workloads and understaffing. However, challenges like side effects, migration of the implant, stakeholder buy-in and inconsistent training hampered the roll-out of the contraceptive implant. For PrEP implants, HCWs preferred long-acting products that were palpable and biodegradable. Our findings highlighted that the characteristics of PrEP implants that are perceived to be beneficial by HCWs may not align with that of potential users, potentially impacting the acceptability and uptake of PrEP implants. Further our data highlight the need for sustained and multi-pronged approaches to training HCWs and introducing new health technologies into communities. Finding a balance between the needs of HCWs that accommodate their heavy workloads, limited resources at points of delivery of care and the needs and preferences of potential users need to be carefully considered in the development of PrEP implants.
**Order of Authors:**	Hilton Humphries, MA (Research Psychology)
	Michele Upfold
	Gethwana Mahlase
	Makhozasana Mdladla
	Tanuja N Gengiah
	Quarraisha Abdool Karim
**Additional Information:**	
**Question**	**Response**
**Financial Disclosure**<br><br>Enter a financial disclosure statement that describes the sources of funding for the work included in this submission. Review	Funding: Funding for the study was provided by South African Medical Research Council Special initiative grant (00251), and partial support from the Department of Science and Innovation-National Research Foundation Centre of Excellence in HIV Prevention.

Powered by Editorial Manager® and ProduXion Manager® from Aries Systems Corporation

the submission guidelines for detailed requirements. View published research articles from *PLOS ONE* for specific examples.<br><br>This statement is required for submission and **will appear in the published article** if the submission is accepted. Please make sure it is accurate.<br><br>**Unfunded studies**<br>Enter: *The author(s) received no specific funding for this work.*<br><br>**Funded studies**<br>Enter a statement with the following details:<br>• Initials of the authors who received each award<br>• Grant numbers awarded to each author<br>• The full name of each funder<br>• URL of each funder website<br>• Did the sponsors or funders play any role in the study design, data collection and analysis, decision to publish, or preparation of the manuscript?<br>• **NO** - Include this sentence at the end of your statement: *The funders had no role in study design, data collection and analysis, decision to publish, or preparation of the manuscript.*<br>• **YES** - Specify the role(s) played.<br><br>* typeset	
**Competing Interests**<br><br>Use the instructions below to enter a competing interest statement for this submission. On behalf of all authors, disclose any competing interests that could be perceived to bias this work—acknowledging all financial support and any other relevant financial or non-financial competing interests.<br><br>This statement is required for submission and **will appear in the published article** if the submission is accepted. Please make sure it is accurate and that any funding sources listed in your Funding Information later in the submission form are also	The authors have declared that no competing interests exist.

declared in your Financial Disclosure statement.<br><br>View published research articles from *PLOS ONE* for specific examples.<br><br>**NO authors have competing interests**<br><br>Enter: *The authors have declared that no competing interests exist.*<br><br>**Authors with competing interests**<br><br>Enter competing interest details beginning with this statement:<br><br>*I have read the journal's policy and the authors of this manuscript have the following competing interests: [insert competing interests here]*<br><br>* typeset	
**Ethics Statement**<br><br>Enter an ethics statement for this submission. This statement is required if the study involved:<br><br>• Human participants<br>• Human specimens or tissue<br>• Vertebrate animals or cephalopods<br>• Vertebrate embryos or tissues<br>• Field research<br><br>Write "N/A" if the submission does not require an ethics statement.<br><br>General guidance is provided below. Consult the submission guidelines for detailed instructions. **Make sure that all information entered here is included in the Methods section of the manuscript.**	The protocol, questionnaires, informed consent forms and study related materials were reviewed and approved by the University of KwaZulu-Natal Biomedical Research Ethics Committee (BE477/19) and the South African National Department of Health Ethics Committee (KZ_201910_017). All participants provided written informed consent prior to being enrolled.

Powered by Editorial Manager® and ProduXion Manager® from Aries Systems Corporation

**Format for specific study types**<br><br>**Human Subject Research (involving human participants and/or tissue)**<br>• Give the name of the institutional review board or ethics committee that approved the study<br>• Include the approval number and/or a statement indicating approval of this research<br>• Indicate the form of consent obtained (written/oral) or the reason that consent was not obtained (e.g. the data were analyzed anonymously)<br><br>**Animal Research (involving vertebrate animals, embryos or tissues)**<br>• Provide the name of the Institutional Animal Care and Use Committee (IACUC) or other relevant ethics board that reviewed the study protocol, and indicate whether they approved this research or granted a formal waiver of ethical approval<br>• Include an approval number if one was obtained<br>• If the study involved *non-human primates*, add *additional details* about animal welfare and steps taken to ameliorate suffering<br>• If anesthesia, euthanasia, or any kind of animal sacrifice is part of the study, include briefly which substances and/or methods were applied<br><br>**Field Research**<br><br>Include the following details if this study involves the collection of plant, animal, or other materials from a natural setting:<br>• Field permit number<br>• Name of the institution or relevant body that granted permission	
**Data Availability**<br><br>Authors are required to make all data underlying the findings described fully available, without restriction, and from the time of publication. PLOS allows rare exceptions to address legal and ethical concerns. See the PLOS Data Policy and FAQ for detailed information.	No - some restrictions will apply

Powered by Editorial Manager® and ProduXion Manager® from Aries Systems Corporation

A Data Availability Statement describing where the data can be found is required at submission. Your answers to this question constitute the Data Availability Statement and **will be published in the article**, if accepted.<br><br>**Important:** Stating 'data available on request from the author' is not sufficient. If your data are only available upon request, select 'No' for the first question and explain your exceptional situation in the text box.<br><br>Do the authors confirm that all data underlying the findings described in their manuscript are fully available without restriction?	
**Describe where the data may be found in full sentences. If you are copying our sample text, replace any instances of XXX with the appropriate details.**<br><br>• If the data are **held or will be held in a public repository**, include URLs, accession numbers or DOIs. If this information will only be available after acceptance, indicate this by ticking the box below. For example: *All XXX files are available from the XXX database (accession number(s) XXX, XXX.*).<br>• If the data are all contained **within the manuscript and/or Supporting Information files**, enter the following: *All relevant data are within the manuscript and its Supporting Information files.*<br>• If neither of these applies but you are able to provide **details of access elsewhere**, with or without limitations, please do so. For example:<br><br>*Data cannot be shared publicly because of [XXX]. Data are available from the XXX Institutional Data Access / Ethics Committee (contact via XXX) for researchers who meet the criteria for access to confidential data.*<br><br>*The data underlying the results presented in the study are available from (include the name of the third party*	The data that support the findings of this study are available from CAPRISA but restrictions apply to the availability of these data, which were used under license for the current study, and so are not publicly available. Data are however available from the authors upon reasonable request and with permission of CAPRISA (data request form available at https://www.caprisa.org/).

and contact information or URL).<br>• This text is appropriate if the data are owned by a third party and authors do not have permission to share the data.<br><br>* typeset	
Additional data availability information:	

Powered by Editorial Manager® and ProduXion Manager® from Aries Systems Corporation

Manuscript

Click here to access/download;Manuscript;Final_CAPRISA 109 PHC paper_16 August 2021_Submission_PlOS.docx

Full Title: Implants for HIV prevention in young women: Provider perceptions and lessons learnt from contraceptive implant provision

Short Title: Implants for HIV prevention in women: Provider perceptions and lessons learnt from contraceptive implant provision

Author List: Hilton Humphries[1]*, Michele Upfold[1], Gethwana Mahlase[1], Makhosazana Mdladla[1], Tanuja N. Gengiah[1] and Quarraisha Abdool Karim[1]

[1]Centre for the AIDS Programme of Research in South Africa (CAPRISA), Nelson R Mandela School of Medicine, University of KwaZulu-Natal, Durban, South Africa

[*]**Corresponding Author**: Hilton Humphries

Mailing Address: Doris Duke Medical Research Institute,

Nelson R Mandela School of Medicine,

University of KwaZulu-Natal, Private Bag X7,

Congella, 4013,

South Africa

Contact Number: +27 31 655 0676

Email: Hilton.humphries@caprisa.org or hilthumphries@gmail.com

Abstract

Preventing new HIV infections, especially amongst young women, is key to ending the HIV epidemic especially in sub-Saharan Africa. Potent antiretroviral (ARV) drugs used as pre-exposure prophylaxis (PrEP) are currently being formulated as long-acting implantable devices, or nanosuspension injectables which release drug at a sustained rate providing protection from acquiring HIV. PrEP as implants (PrEP Implants) offers an innovative and novel approach to expand the HIV prevention toolbox. Feedback from providers and future users in the early clinical product development stages may identify modifiable characteristics which can improve acceptability and uptake of new technologies. The perspectives of Health Care Workers (HCWs) ~~HCWs perspectives~~ and lessons learned during the rollout of contraceptive impants will allow us to understand what

factors may impact the roll-out of PrEP implants. We conducted eighteen interviews with HCWs (9 nurse and 9 community healthcare workers) in rural KwaZulu-Natal, South Africa. HCWs listed the long-acting nature of the contraceptive implant as a key benefit, helping to overcome healthcare system barriers like heavy workloads and understaffing. However, challenges like side effects, migration of the implant, stakeholder buy-in and inconsistent training on insertion and removal hampered the roll-out of the contraceptive implant. For PrEP implants, HCWs preferred long-acting products that were palpable and biodegradable. Our findings highlighted that the characteristics of PrEP implants that are perceived to be beneficial by HCWs may not align with that of potential users, potentially impacting the acceptability and uptake of PrEP implants. Further our data highlight the need for sustained and multi-pronged approaches to training HCWs and introducing new health technologies into communities. Finding a balance between the needs of HCWs that accommodate their heavy workloads, limited resources at points of delivery of care and the needs and preferences of potential users need to be carefully considered in the development of PrEP implants.

Introduction

Preventing new HIV infections remains a global challenge and a major barrier to achieving the United Nations 2030 goal of ending AIDS as a public health threat. Recent developments such as oral pre-exposure (PrEP) and the vaginal ring provide options that, for the first time, offer a women-initiated HIV prevention method(1–4). While these provide valuable protection against HIV infection, some young women aged 15-24 years old in sub-Saharan Africa (where 25% of global new infections occur) face challenges with adherence(5). The PrEP landscape is rapidly expanding to address these adherence challenges by developing different long-acting slow-release products(6). The array of new PrEP products under clinical development mirrors available biomedical pregnancy prevention methods? family planning providing an opportunity to gain insights on provider and target population product preferences based on provider and user experiences of contraceptive options currently being used.

The 3-year, single rod etonogestrel contraceptive implant, Implanon NXT®, has been available through the public and private health care system in South Africa since 2014(7–13). Initial high uptake through public health care clinics was observed(7) but myths about the implant(14,15), systemic side effects, low levels of user knowledge, inadequately trained providers and inadequate community engagement contributed to a steady decline in the uptake and continued use of Implanon NXT(7–13). Lessons and attitudes towards the contraceptive

implant may offer insight into the design and roll-out of subdermal implants for HIV prevention. Antiretroviral drug used as pre-exposure prophylaxis (PrEP) are currently in development to be formulated into subdermal implants, either routinely removable or biodegradable or injectable nanosuspensions that release anti-HIV drug at a sustained rate and provide protection from acquiring HIV. Thus, PrEP as implants (PrEP Implants) offer a new technology to expand the HIV prevention toolbox. A An implant containing tenofovir for PrEP HIV prevention ~~PrEP implant containing tenofovir alafenamide (TAF) for HIV prevention~~ is currently in the early stages of clinical development(16).

Challenges with the acceptability of new HIV prevention technologies mean ~~means~~ that end-user and future-provider research at the early stages of clinical product development are ~~is~~ critical. Health care workers (HCWs) act as ~~are~~ important gatekeepers to the promotion of new health technologies, providing a critical view ~~an important view~~ of the challenges to ~~that face~~ the uptake and implementation of new technologies within the public health care setting(17). Previous research in urban/peri-urban areas of South Africa highlighted that the design and duration of coverage of PrEP implants were important to HCWs, especially for overcoming barriers ~~to overcome barriers~~ within health systems like staff shortages and capacity development(17). As with potential users, when ~~if~~ HCWs are engaged as part of the development process about modifiable aspects of new products they may be more likely to promote them when they are licensed and available programmatically(18). Additionally, HCWs can be engaged to contribute to improvements in messaging, information and modifiable aspects of the PrEP implants in a manner that is responsive to the needs of future users and improves the provider experience.

suggestion: define modifiable in this context

I think that it is more accurate to say that by engaging the potential user and service providers, you can then ensure that the products are responsive to the factors you mention related to utilization, and thus, it will be more likely that there is acceptability. The way this is phrased it seems like the acceptability has to be responsive; where it should be that acceptability is contingent on the responsiveness of the product.

Engaging potential users and service providers ensures that the acceptability of products responds to the social and contextual factors in which they will be used. This moves us beyond a product efficacy focus to developing products that fit practically into the context and life of those who will use or provide them. This manuscript describes, or examines ~~reports~~ on the perspectives of HCWs ~~who are~~ providing contraceptive implants or information about contraceptive implants in rural KwaZulu-Natal. We explore their insights into the design and contextual issues that may act as challenges and facilitators to the use of PrEP implants for HIV prevention amongst young women living in a high HIV burden rural community in South Africa.

Materials and Methods

Study Community

Vulindlela is a rural community situated 150km west of Durban, KwaZulu-Natal, South Africa. It has a high burden of HIV with an estimated antenatal prevalence of over 40% in women aged 20-24 years(19), and a population prevalence of 36.7%(20). Access to health care is through nine primary health care clinics (PHCs). Outside of these PHC clinics, mobile clinics and community health care workers (CHWs) provide additional health care support within the community.

Procedure

Data were collected between October 2019 and March 2020. Purposive sampling was used to identify a professional nurse and a CHW at each of the nine PHC clinics in the study community. We included CHWs as they provided an important link between the formal health care system and the community(18). A participatory approach was used to develop the in-depth interviews (IDI) guide that included discussions with local community partners including the CAPRISA Research Support Group to ensure conceptual and linguistic integrity in the local language (isiZulu). Following written consent, individual IDI's using a semi-structured interview guide were completed at a time convenient to the participant. All interviews were conducted by experienced interviewers who are fluent in English and isiZulu. The IDI guide included items to ascertain participant knowledge and experiences with the contraceptive implant; challenges and successes of past and present provision of the contraceptive implant; their views on the potential of implants for HIV prevention; and how they would introduce it to potential users and/or create demand and facilitate uptake in young women. Each interview lasted approximately one hour. All IDIs were conducted in isiZulu and were audio-recorded, transcribed and then translated into English. English transcripts were quality checked by a research assistant fluent in isiZulu and English against the isiZulu interview recordings for accuracy. Participants were offered refreshments but no reimbursement for their time. Approval for the study was obtained from the University of KwaZulu-Natal Biomedical Research Ethics Committee (BE477/19) and the South African National Department of Health Ethics Committee (KZ_201910_017).

Data Analysis

An iterative process was used to analyse the data. After conducting a literature review and knowledge from the study team's prior experience ~~in~~ integrating tenofovir gel into family planning services(21), preliminary themes were included in a draft codebook *a priori*. These

were then validated by reviewing a sub-set of transcripts(22). Through multiple independent reading and re-reading of a sub-set of transcripts, themes and codes were reviewed until consensus about the representativeness of the theme was reached by the primary research team. Themes identified were documented following guidelines by Boyatzis(23). The consensus codebook was then applied to the full data iteratively to identify any new themes that needed to be added(24). Once the coding framework was finalised, coding was undertaken using NVivo-12 (QSR International). This final coding was done independently by four members of the research team, followed by group meetings to reach consensus on themes and codes and continued improvements on inter-rater reliability and adaptations to the codebook. Once inter-rater testing was complete, the themes were used as a basis for analysis and discussion of the overall research question.

Results

Demographics

A total of eighteen interviews were completed, nine with PHC nurses and nine with CHWs. All the participants were female. The mean age of the PHC nurses was 41 years (range: 32 - 48 years), and 41 years for CHWs (range: 30 - 49 years). All those interviewed had over 3 years of experience working in public health settings, and had experience with providing information about Implanon NXT and discussing contraception with potential users. Details about the experience with inserting and removing Implanon NXT and training received to do so for PHC nurses is detailed in Table 1.

insertion and removal training?

Is this number of years of experience with Implanon defined as number of years since training? Providing insertion/ removal? Just the number of years providing info and contraceptive discussions? Any? Definition of "experience" in this context needed

Table 1: Training Details for PHC Nurses working in rural KwaZulu-Natal

**Years' experience with Implanon**		**Number Implanon inserted**		**Number Implanon removed**		**Training on Implanon**		**Received Supervision and mentorship**	
(n=9)		(n=9)		(n=9)		(n=9)		(n=9)	
<2	11% (1)	0	11% (1)	0	11% (1)	No training	11% (1)	**Yes**	55.6% (5)
3-5	63.6% (7)	1-10	22.2% (2)	1-10	44.4% (4)	Theoretical	11% (1)	**No**	33.3% (3)
>5	11% (1)	>10-100	33.3% (3)	>10-100	44.4% (4)	Theoretical and practical	44.4% (4)	**Not applicable**	11% (1)
		>100	33.3% (3)	>100	0% (0)	Trained by a colleague	33.3% (3)		

The analysis identified four major themes, 1) Benefits and challenges associated with contraceptive implants, 2) Important characteristics for an HIV prevention implant, 3) Capacity development and training of service providers, and 4) Engaging the local community on new

technologies (Table 2). For the extracts, note the following conventions: I= interviewer, N=Nurse and C=Community Healthcare Worker.

Table 2: Outline of major themes identified during analysis

Major Theme	Sub-Themes
Benefits and Challenges of the Contraceptive Implant that impact Acceptability<br>**Why are some major themes capitalized and not others?**	Long-acting: Important theme amongst all HCWs. The theme dealt with the benefit that the long-acting contraceptive implant afforded the user because it 1) it did not rely on frequent user adherence, 2) allowed for less pain due to reduced frequency of administration, and 3) improved user convenience
	Side Effects: A theme characterising the importance of side-effects amongst contraceptive implant users. Highlights side-effects as a key barrier to contraceptive implant acceptability.
	Migration of the Contraceptive Implant: the theme that deals with concerns over the migration of the implant away from the insertion site. Important theme as impacted the development of myths about the contraceptive implant.
	The Social Context: Important gatekeepers: Theme that highlight the importance of key stakeholders for contraceptive use acceptability amongst women.
Important characteristics for an HIV prevention implant	Implant Size and Duration of drug efficacy: This theme deals with the preferred size and period of protection that HCWs feel would offer the highest acceptability amongst potential users.
	Palpability: this theme deals with the importance of palpabilty for improved ability to feel and remove the implant. Sub-themes of discretion concerns and issues of adherence and links to biodegradability are also included.
	Biodegradability of implant: this theme deals with the HCWs perception of biodegradability as a benefit to user acceptability.
Capacity development of service providers to provide implants	This theme highlights the training and capacity development that HCWs received during the roll-out of the contraceptive implant and lessons we must learn for future training on new technologies such as the PrEP implant.
Engaging the local community on new technologies	This theme highlights the importance of community engagement, the challenges and limitations of previous community engagement for the contraceptive implants. It highlights lessons learned for future rollout of new technologies like the PrEP implant.

Benefits and Challenges of the Contraceptive Implant that impact Acceptability

As the characteristics of the contraceptive implant provide important insight into the factors that may similarly impact PrEP implants, this theme looks at how the HCW experience of benefits and challenges of the contraceptive implants may have impacted acceptability amongst young women. Sub-themes include long-acting, side effects, migration of the contraceptive implant and the social context: important gatekeepers. These are discussed below:

Long-acting nature of the contraceptive implant

Across nurses and CHWs, the long-acting nature of the contraceptive implant was provided as its primary benefit. This was because 1) it did not rely on frequent user adherence (daily or 2 to 3 monthly), 2) less frequent provision meant less frequent pain, and 3) long-acting meant more user convenience and the benefits of saving the user both time and money:

> *I see a lot of benefits, mainly time-saving, as I don't need to [come] to the clinic all the time. It saves both time and money. Also, if I feel pain I would feel it once and the next time I feel pain will be after three years when the implant is removed unlike feeling pain every 3 months. (IDI3 – Nurse)*

Additionally, both CHWs and nurses alike emphasised that clinics were often understaffed and under-resourced. The long-acting nature of an implant was a benefit for reducing the burden on HCWs and on potential user's time:

"*Isn't some [users] are employed, so they will not have to frequently request day-offs causing employers to complain, and those that are studying don't have to miss any classes. So three years is very convenient, she would only return after three years to the clinic" (IDI13– Nurse)*

Thus, the long-acting nature of an implant may have two-fold benefits; it reduces the work burden of the clinics and reduces the time burden on potential users. However, nurses highlighted that a PrEP implant would still impact clinic workloads as a user would still need to undergo regular HIV testing saying, *"A person [should] use the implant for at least a year and that person should get tested [for HIV] again and be found to have not been infected"*.

Side Effects of the contraceptive implant

Across all the interviews side effects were the primary critical barrier to the continued use of contraceptive implants. The side effects experienced appeared to have the greatest effect on long-term use or continued use of contraceptive implants. As one CHW reported:

> *I know [two people] who stayed [adherent] even when they were having non-stop bleeding but they did stay for 3 years only [...], then said they [would] not re-insert after removing it (IDI19_CHW)*

One CHW who had used the contraceptive implant provided insight into the side effects she experienced. These motivated her early removal of the implant:

> *I used the Implanon myself but it did not treat me well. I was dizzy, had headaches, pains and I had problems with heavy bleeding. I was bleeding and had excruciating pain- period pains that I had never had before. When I was about to menstruate it felt like a rock was going through me. (IDI15_CHW)*

These side effects may be enough to discourage use amongst women, and may become a reason why women choose to remove implants regardless of whether or not they experience the side effect themselves:

> *They complain of heavy bleeding but sometimes when we check some of them they use that as an excuse it is not that they are really having heavy bleeding, maybe they just don't want it. (IDI7_Nurse)*

The experience of side effects is critical to decision-making and acceptability. Side effects, experienced or a product of social learning (learning through observation), may serve to discourage use regardless of efficacy. While side-effects are primarily a result of the active drug and not the device, the PrEP implant side effect profile will be critical to future acceptability and should form a part of future community messaging development.

Howver - in this context, one of the major side-effects that resulted in dissatisfiaction or not wanting to reinsert is menstrual/ uterine bleeding and dicsomfort/pain. If PrEP implant is not combined with hormonal contracpetive, is there a reason to think that this particular side-effect will play a role somehow in uptake and/or acceptbaility?

Migration of the Contraceptive Implant

The movement of the contraceptive implant from its original position was a concern for users, as noted by a nurse:

> *I have experienced it because so many ladies have come to remove the implants because they were not on the same side where they had been inserted initially. Normally there's a specific position in which you put them, but they would relocate, whilst others (implants) would relocate and they wouldn't feel it, then it would have to be removed in [surgical] theatre. (IDI4_Nurse)*

The potential for movement of the implant from the original insertion area becomes the basis for myths and concerns about the implant travelling into other organs(25,26) and highlights the importance of practical training on correct insertions procedures. The issue of

migration of the implant was closely linked to the issue of palpability and biodegradability as well.

The Social Context: Important gatekeepers affecting uptake of contraceptive implants

The decision to use implants takes place within the broader social context in which a person lives. Both nurses and CHWs noted that acceptance by certain gatekeepers' (like partners and church membership) impacts whether a woman decides to use the contraceptive implant. Partners will affect implant uptake, a CHW noted that young women may "*end up being scared that their partner will kill them because they have an implant and the partner wants a child." (IDI15_CHW)*. Other stakeholders such as churches may further influence product use, as noted by one nurse:

> *Some men were very strict and refused for their women to have an Implant inserted, particularly those that belong to [...] religion. They would say that it is not allowed in their religion and any form of contraception is not allowed. (IDI4_ Nurse)*

Social influences, especially partners and important social institutions are powerful players for influencing the uptake and acceptability of sexual and reproductive health services and new HIV technologies amongst women. The effective rollout of new products requires the *continued* engagement of several different stakeholders as it will impact on whether products are used.

Important characteristics for an HIV prevention implant

HCWs had clear opinions about what they thought the implant for HIV prevention should look like to improve acceptability amongst potential users. Importantly, opinions were highly congruent across both nurses and CHWs. Broadly, HCWs spoke about three key areas: 1) Implant size and duration of drug efficacy, 2) Palpability and 3) Biodegradability of the implant.

Implant Size and Duration of drug efficacy

Overall, most HCWs felt that the PrEP implant should be the same size as the current contraceptive implant or smaller. Some CHWs felt that a PrEP implant slightly larger than the contraceptive implant would be acceptable if this larger size coincided with a longer-acting product:

I think the big rod that lasts for [a] longer period will be much better, because as much as people come to clinics but they are sometimes reluctant because clinics are always full. (IDI17_CHW)

All but one HCWs felt that the implant should last for a minimum of one year, but two years or more was ideal to *"delay [users] coming to the clinic"*. One concern raised by a CHW highlighted that long-acting products could stop users from checking if the product was still active and working properly, and suggested that regular check-ups should be implemented saying "*It should not last for a long time, there should be a period where they can check if it is still working…" (IDI15_CHW).*

The length of time the drug remained efficacious was not the only important factor. Perceived efficacy or lack thereof could be a barrier to uptake. Three HCWs relayed experiences where patients fell pregnant while using a contraceptive implant and this fuelled discouraging myths about the product and its efficacy:

I think the other thing that made others [potential users] not to be interested is that they have heard the others telling them that I [had the implant] inserted then after 3 months, I became pregnant. (IDI12– Nurse)

This highlights important considerations for an HIV prevention implant. Careful messaging around how HIV prevention products reduce but do not eliminate the risk of HIV infection will be critical to ensure that users are not disappointed by their expectations of efficacy versus the actual efficacy of a product to prevent HIV infections.

Palpability

Overall, most nurses and CHWs interviewed felt that the PrEP implant should be palpable so that it could be felt and easily removed. Lessons from the contraceptive implant meant that HCWs felt that because palpability may be equated with safety (i.e. it had not migrated) and product efficacy (i.e. because if a user can feel it, they think it is working), young women would want to feel the implant. As one nurse said:

I think [being] palpable will be good. [The] first reason why I'm saying that [is that] even the owner will be sure it is still there. Another reason [is that they] will feel that I'm still safe, because once it disappears even the patient will have that doubt, "Is it still working or is [it] going to damage one of my organs?" (IDI2_Nurse)

However, one caution raised by CHWs was that palpability may negatively affect the discretion of the implant to safeguard young women against unwanted harassment and violence from partners. As learned from the use of contraceptive implants, a CHW noted:

> *[…] I was in a festival and this boy said [that] they pretend to be playful with a girl by touching her arm, in order to confirm if she has the Implanon in her arm. My question is, is there a way for it to be placed in an area where a person will not be able to see it so that it can be hidden, for males not to know that there is an implant there. Because they will end up being scared that their partner will kill them because they have an implant and the partner wants a child. (IDI15_CHW)*

This highlights a potential disconnect between what is a benefit for an HCW versus a potential user. Being palpable may have benefits for HCWs as it eases the removal of an implant, but for discretion, palpability may serve as a challenge to uptake amongst potential users. It also highlights an area of potential user conflict where the need for discretion will be weighed against the belief that palpability equates with continued efficacy and as a means to confirm the implant has not migrated.

Biodegradability of Implant

Perceptions of a biodegradable implant were largely positive from both nurses and CHWs and interlinked with the sub-theme of palpability. Most HCWs felt that a biodegradable implant would result in less pain as it would avoid the need for removal, and result in less scarring which has been a problem with the contraceptive implant. This would make the PrEP implant more acceptable:

> *I: Which would be better for this HIV one – […] would it better that they come remove it or that it is absorbable? Just like stitches are absorbable.*
> *N: In my opinion, I think it should be absorbable because it is easy to do the insertion, but when removing it, [that is] when it results in scarring which no one likes. (IDI13 – Nurse)*

An area of ambivalence was the impact that a biodegradable PrEP implant could have on adherence. Supporting adherence, a CHW noted that the disappearance of the PrEP implant may serve as a reminder to have it replaced, as the user would equate the disappearance with reduced efficacy and hence the need to have it replaced: "*I think it's better to be biodegradable because if I have [to] remove it at the clinic, I will have this [implant efficacy] in my heart that*

it is still effective because I can feel it's there". However, this same benefit could serve to negatively affect adherence and ultimately its prevention effectiveness because "*[removing an implant at a] clinic [may remind users] of the duration of the implant because sometimes people forget their regular visits, whereas when it is biodegradable, they would not bother going to the clinic to get another one'' (IDI9_CHW).*

HCWs raised that biodegradability may raise concerns about where the implant goes when it biodegrades. One nurse switches her answer when contextualising the implementation of a biodegradable implant in her community, in relation to her experiences providing contraceptive implants:

> *I think absorbable is ideal. […]But maybe they will ask - the client will ask about where it is going when it is absorbed because, they are reluctant to most of the things […] even with the no period thing of Implanon, they are curious about where the blood is going if you do not see your periods. So [being] absorbable will raise questions with the community. It is better to remove it so we can show it to them (IDI16 – Nurse)*

These concerns highlight that while biodegradability may have benefits for HCWs who have high workloads; this option may have a complex impact on community acceptability.

Capacity development of service providers to provide implants

The continued capacity development of HCWs to provide information about and then implement new technologies is critical. Many nurses and CHWs reported that they had received at least once-off training, however, only nurses reported receiving formal and limited mentoring. As one nurse stated:

> *I: And eh, have you received any mentorship?*
> *N: Yes.*
> *I: Yes. From who?*
> *N: From (Clinician A), because when I came back from the training I started having problems because I was not used [to it], I was not skilled enough to do it.*
> *I: Okay. And how many days or how many hours?*
> *N: It was, no, it was… maybe three hours.(IDI16_Nurse)*

In most instances, training did not happen for all nurses. Rather a train-the-trainer system whereby training was done for a single nurse or a couple of nurses, who then trained the remainder of the clinic staff was implemented. This causes problems for long-term service provision when staff rotation or attrition occurs, and new staff need to be trained:

I: Did all the nurses do the Implanon training?
N: No.
I: How were you chosen to be part of the implant insertion training?
N: The ones that were trained previously by another facility [training session] left the clinic. The other one is new and I think it depended on one's interest because some did not even attend the briefing that was done by the nurse who attended the training. (IDI7_Nurse)

Training appeared to be inconsistent for CHWs. The quality of the CHW training appeared dependent on the supervisor who was responsible for the CHWs and could vary from a five-day-long training to no training at all. As noted by a CHW:

C: No I haven't attended any training. I am using the information that I have overheard in the clinics.
Interviewer: Okay. So the nurses didn't give you any training so that you will be able to inform the people well about the implant?
C: No. We depend on the information that we have overheard about the implant. (IDI11_CHW)

Training and plans for continued training of staff, especially those that interface directly with potential users, will be critical to the rollout of future PrEP implants.

Engaging the local community on new technologies

Similar to the inconsistencies in the level and quality of training, community engagement activities at the start of new campaigns seemed to depend on the clinic and the resources available to them. In the first extract the nurse highlights that the clinic engagement occurred through multiple teams:

I: The community where you were working at [community name], was the community ever engaged before this method [Contraceptive Implant] was introduced to say this what we'll be introducing just to engage the community?
N: Yes, because we normally do health education in the mornings, I would say yes. Because there are also school nurses that do that and the school health team.
I: They go out to the community?
N: Yes, yes.
I: How do they do it, do they go house to house?
N: Family health go[es] house to house, school nurses' school to school, mobile [clinics] go to certain points and us at the clinic. (IDI4_Nurse)

Then, a CHW at another clinic highlighted that outreach was limited to talks in the clinic only, with no outreach in the community:

C: The community was engaged because it was also announced on radio and on television during the NEWS time. The counsellors did educate the people who came to the clinic in the morning about this contraceptive implant.
I: So there was no outreach campaign on implants?
C: Not that I know of.(IDI9_CHW)

Some HCWs reported that with the rollout of PrEP implants, there should be improved coordination amongst different stakeholders to ensure that engagement plans are coordinated and that information is consistent. A CHW highlighted *"I would like the Department of Health to involve all health employees and NGOs in order to discuss a way forward. After that, when they have decided that it will be introduced [we can move forward]"*. In addition, a nurse interviewed highlighted the importance of integrating services into schools to target young people, highlighting that:

I think it would be good if we were to go to schools for the [contraceptive] insertions because the younger ones do not want to come to the clinic. They are the ones who are the target. It is difficult for them to come to the clinic because they come out of school late. They come to the clinic late and then they have to leave late or else they have to start here and delay going to classes. (IDI16_Nurse)

The findings highlight the inconsistency in engagement plans with potential users and the communities in which the user will use them. Systematic, sustainable programmes are needed to ensure long-term community engagement with new HIV prevention technologies.

Discussion

Young women in sub-Saharan Africa remain the most vulnerable to HIV infection and expanding our toolbox of HIV prevention options is critical to achieving epidemic control. Health care workers play an important role in ensuring that efficacious health products are accessed and utilised by potential beneficiaries and end-users(17). They offer an invaluable perspective on how communities will respond to the introduction of new technologies and should play an important role in early clinical product development, and driving the acceptability and uptake of these products as they become available. Lessons from the rollout of contraceptive implants provide important insights into the factors that may impact the acceptability of PrEP implants.

Likely, many of the benefits and challenges that characterise the contraceptive implant will similarly affect PrEP implants(7,17,27,28). For HCWs, especially the nurses, the benefits of a long-acting PrEP implant were explicitly linked to its impact on the running of the clinic and managing workloads: products that reduce visit frequency and are palpable and flexible to make removal easier. However, these preferences highlight complex issues that go beyond a tick-box of key characteristics that a PrEP implant should have, and highlight that tensions may exist between the preferences of HCWs and how this translates to impact potential users.

Similar to research in other parts of South Africa(17,29), and consistent with the benefits of contraceptive implants, both nurses and CHWs had a preference for long-acting products. However, while having a long-acting product (> 1 year) was preferred and considered a major benefit of the current contraceptive implant, for HIV prevention, the implant may not reduce workloads as significantly as hoped. For HIV prevention, long-acting products will not negate the need for regular HIV testing to ensure that a client has not seroconverted, and is timeously referred to HIV treatment services. Thus, the introduction of these technologies is likely to require clients to still come to clinics regularly and as the implant will likely be available to both men and women it may increase the numbers seeking these services. Further, while long-acting products offer long-term protection, user expectations of protection against HIV infection will need to be managed. Limits to how efficacious a product needs to be easily

and carefully explained to ensure that users are not disappointed by their expectations of efficacy versus the actual efficacy of a product to prevent HIV infections.

Palpability was considered important to both nurses and CHWs for different reasons. Nurses felt that palpable PrEP implants made it easier to find and remove, while CHWs felt it would help users be certain that the implant had not moved, and that users would associate its presence with the fact that it was still working. On the negative side, palpability may have the unintended consequence of reducing discretion and invisibility with partners or family members, a major benefit of injectable long-acting HIV prevention options(17,30). As partners have a significant influence on the use of current HIV prevention technologies(31,32), users of PrEP implants will weigh concerns about discretion against the perceived benefits of the implant to reduce HIV infection, mediated by their risk perception. So, while palpability may serve to make insertion and removal easier for nurses, it may negatively influence the acceptability amongst users, especially female users. Consideration of where an implant is placed on the body and how social relationships will affect sexual health decisions will be critical. An issue that requires exploration with potential users is whether using the PrEP implant could become a "badge of honour", being equated with an HIV negative status. However, there is the risk that partners may still assume the PrEP implant is a contraceptive implant which could cause problems if the partner wants to have children. Careful messaging designed to explain the differences between the contraceptive and PrEP implant will need to be developed to ensure that the challenges associated with one product are not conflated with the other. More research about how partners and family members will influence the uptake of PrEP implants is needed to understand the impact these important social connections will have on the uptake, especially if PrEP implant discretion is limited.

Biodegradability had similar important implications for acceptability. Our data confirm previous research, which found that most HCWs (both nurses and CHWs) are in favour of an implant that would biodegrade(17). The motivation for biodegradability was linked to reducing workloads, a lack of capacity to remove implants and minimise the pain associated with removal for users(17,33). However, biodegradability may give rise to community myths similar to those found in the contraceptive literature(25,34). A lack of understanding about where the product disappears too, concerns about how it may affect the body and whether other potential unrelated health issues will be attributed to the disappearance of the implant will all serve to undermine potential acceptability regardless of its benefits for the health care system. HCWs also raised concerns that the disappearance of the product may be associated with

reduced palpability and hence perceived efficacy which may lead to concerns amongst potential users that they are no longer protected. While some felt that this may positively impact adherence (reminding users to get a new one) others felt that the slow biodegrading implant could negatively impact adherence because users would not have the reminder of a palpable implant in their arm. This raises an opportunity for modifiable characteristics of implant technologies within the clinical development phase. It highlights that within the development of the same products, broadening the range of the product characteristics available may serve to help fit the product to the needs and preferences of the user more specifically.

An important lesson from the roll-out of the contraceptive implant was that side effects (especially heavy bleeding) were a key reason for poor user uptake and early removal which may in turn fuel myths and social learning which deter other potential users(17,28,31).While side effects may be attributed to the active drug used in the implant rather than the delivery method, careful consideration of the side-effect profile on PrEP implants will be critical to real-world usability. Negative side-effects have been a key factor impacting adherence to ARV medication(35) as well as other available HIV prevention technologies, like oral PrEP(29). Therefore community education about the differences between the contraceptive implant and PrEP implant will be critical to avoid negative associations with the contraceptive implant being conflated with those of the PrEP implant. Careful, and transparent disclosure about possible side effects and how these differ from other available HIV prevention technologies will be an important part of the messaging and community engagement processes that need to occur. The experience of side effects will likely be critical to the decision-making of users regardless of product efficacy.

Training and community engagement form the pillars of developing sustainable healthcare systems that can respond to the needs of communities. Similar to contraceptive implants, the capacity of health care workers and lack of long-term sustainable plans for practical training and retraining staff (especially on insertions and removals) may negatively impact PrEP implant provision(7,17,36). Sustainable models for mentorship and practical training of HCWs will be needed to ensure the successful rollout of new technologies. In addition, multi-pronged, high quality, and sustained community engagement will be critical to the rollout of PrEP implants. Without empowered and knowledgeable providers and communities, or sustained programmes for engagement, it will be easy for misinformation to spread, and for the initial excitement for new products to fade. HIV prevention technologies, like the PrEP implant, may benefit from the contraceptive implant in terms of acceptability

because it will be available to both men and women. The engagement should be broad-based, targeting multiple stakeholders including male and female partners, parents, and social stakeholders such as schools and religious institutions. While using a PrEP implant may be inherently an individual choice, the decision to use it will be influenced by a complex interplay of other factors such as community acceptance, religious affiliation, and partner approval, and broad-based community engagement and promotion of use amongst both men and women should be done from the start. While an PrEP implant may not be acceptable for all users, any long-term acceptability will need to target multiple stakeholders to ensure that the product does not become irrelevant because a user is unable to overcome the various challenges to safe and discrete use in the real world.

One thing to consider as part of limitations is the bias of the Nurses and HCWs in your sample - they are all women themselves, and may be within their reproductive years. They themselves may have their own perspectives on the implant from their own personal (not professional) experiences with the contraceptive implant. This is beyond the scope of your study but should be addressed as a source of possible bias in your findings. Also possible difference by either Nursing profession or HCW profession, given difference in education level attainment.

Our study has some limitations. The sample size is small and situated in a specific rural geographic location, limiting the representativeness and generalisability of our findings. However, given the diversity of epidemics within and between countries a localised understanding is critical to know your epidemic and breaking chains of transmission. However, the findings are consistent with other studies conducted in different parts of South Africa(7,17,37). The findings are formative and need to look at how HCW opinions will differ from that of potential users. Research investigating how these findings may be different for potential users is underway. Additionally, research on community engagement strategies and the development of lexicons for promoting the use of new technologies amongst different age groups and user groups will be needed.

Our findings highlight that the characteristics of PrEP implants that are perceived to be beneficial by HCWs may not align with that of potential users. These provider identified characteristics have to be carefully considered and weighed up against potential user preferences and may need to be addressed in provider training to introduce new technologies. Finding a balance between the needs of HCWs that accommodate their heavy workloads, limited resources at points of delivery of care and the needs and preferences of potential users need to be carefully considered. These data highlight the need for sustained and multi-pronged approaches to introducing new health technologies into communities. Complementary social science research, occurring alongside the development of new products can facilitate effective products are able to reach their full potential for HIV prevention.

Acknowledgments

The authorship team would like to thank all the study staff, the CAPRISA Vulindlela

Community Research Support Group, the Vulindlela community, Provincial and District Departments of Health, and the healthcare workers willingness to contribute to and participate in the study. We thank the South African Medical Research Council Special initiative grant (00251), South African Department of Science and Innovation-National Research Foundation Centre of Excellence in HIV Prevention for their funding support. We would also like to acknowledge Brett Marshall and Revina Munsamy, two masters fellows who assisted the team with coding the data.

References

1. Thomson KA, Baeten JM, Mugo NR, Bekker L-G, Celum CL, Heffron R. Tenofovir-based oral preexposure prophylaxis prevents HIV infection among women. Curr Opin HIV AIDS. 2016 Jan;11(1):18–26.

2. Bekker L-GG, Roux S, Sebastien E, Yola N, Amico KR, Hughes JP, et al. Daily and non-daily pre-exposure prophylaxis in African women (HPTN 067/ADAPT Cape Town Trial): A randomised, open-label, phase 2 trial. Lancet HIV. 2017 Feb;5(2):e68–78.

3. Baeten JM, Palanee-Phillips T, Brown ER, Schwartz K, Soto-Torres LE, Govender V, et al. Use of a Vaginal Ring Containing Dapivirine for HIV-1 Prevention in Women. N Engl J Med [Internet]. 2016 Dec;375(22):2121–32.

4. Nel A, van Niekerk N, Kapiga S, Bekker L-G, Gama C, Gill K, et al. Safety and Efficacy of a Dapivirine Vaginal Ring for HIV Prevention in Women. N Engl J Med. 2016;375(22):2133–43.

5. UNAIDS. Women and HIV: A spotlight on adolescent girls and young women. 2019;1–20. Available at https://www.unaids.org/en/resources/documents/2019/women-and-hiv

6. IAVI. Evolving access pathways for long-acting HIV prevention products. 2021. Available from: https://www.iavi.org/phocadownload/Access-pathways-for-long-acting-HIV-prevention-products.pdf

7. Pleaner M, Morroni C, Smit J, Lince-Deroche N, Chersich MF, Mullick S, et al. Lessons learnt from the introduction of the contraceptive implant in South Africa.

South African Med J. 2017 Oct 31;107(11):933.

8. Mullick S, Chersich MF, Pillay Y, Rees H. Introduction of the contraceptive implant in South Africa: Successes, challenges and the way forward. South African Med J. 2017 Sep 22;107(10):812.

9. Dunbar MS, Kripke K, Haberer J, Castor D, Dalal S, Mukoma W, et al. Understanding and measuring uptake and coverage of oral pre-exposure prophylaxis delivery among adolescent girls and young women in sub-Saharan Africa. Sex Health. 2018;15(6):513.

10. Rees H, Pillay Y, Mullick S, Chersich MF. Strengthening implant provision and acceptance in South Africa with the 'Any woman, any place, any time' approach: An essential step towards reducing unintended pregnancies. South African Med J. 2017 Oct 31;107(11):939.

11. Pillay D, Morroni C, Pleaner M, Adeagbo OA, Chersich MF, Naidoo N, et al. Gaps in monitoring systems for Implanon NXT services in South Africa: An assessment of 12 facilities in two districts. South African Med J. 2017;107(10):827.

12. Ralph LJ, McCoy SI, Shiu K, Padian NS. Hormonal contraceptive use and women's risk of HIV acquisition: A meta-analysis of observational studies. Lancet Infect Dis. 2015;15(2):181–9.

13. Morrison CS, Richardson BA, Mmiro F, Chipato T, Celentano DD, Luoto J, et al. Hormonal contraception and the risk of HIV acquisition. Aids. 2007 Jan 2;21(1):85–95.

14. Brown A, Harries J, Cooper D, Morroni C. Perspectives on contraceptive implant use in women living with HIV in Cape Town, South Africa: A qualitative study among primary healthcare providers and stakeholders. BMC Public Health. 2019;19(1):1003.

15. Patel RC, Morroni C, Scarsi KK, Sripipatana T, Kiarie J, Cohen CR. Concomitant contraceptive implant and efavirenz use in women living with HIV: Perspectives on current evidence and policy implications for family planning and HIV treatment guidelines. J Int AIDS Soc. 2017;20(1):21396.

16. Karim SSAA, Abdool Karim Q, Gengiah TN. CAPRISA 018: A phase I/II trial to assess the safety, acceptability, tolerability and pharmacokinetics of a sustained-release tenofovir alafenamide sub-dermal implant for HIV prevention in women. 2019.

Available from: https://www.caprisa.org/Pages/CAPRISAStudies

17. Krogstad EA, Montgomery ET, Atujuna M, Minnis AM, O'Rourke S, Ahmed K, et al. Design of an Implant for Long-Acting HIV Pre-Exposure Prophylaxis: Input from South African Health Care Providers. AIDS Patient Care STDS. 2019 Apr;33(4):157–66.

18. Schneider H, Hlophe H, van Rensburg D. Community health workers and the response to HIV/AIDS in South Africa: tensions and prospects. Health Policy Plan. 2008 Mar 4;23(3):179–87.

19. Kharsany ABM, Frohlich JA, Yende-Zuma N, Mahlase G, Samsunder N, Dellar RC, et al. Trends in HIV Prevalence in Pregnant Women in Rural South Africa. JAIDS J Acquir Immune Defic Syndr. 2015 Nov;70(3):289–95.

20. Kharsany ABM, Cawood C, Khanyile D, Lewis L, Grobler A, Puren A, et al. Community-based HIV prevalence in KwaZulu-Natal, South Africa: results of a cross-sectional household survey. Lancet HIV. 2018 Aug;5(8):e427–37.

21. Mansoor LE, Yende- Zuma N, Baxter C, Mngadi KT, Dawood H, Gengiah TN, et al. Integrated provision of topical pre- exposure prophylaxis in routine family planning services in South Africa: a non- inferiority randomized controlled trial. J Int AIDS Soc. 2019 Sep 11;22(9).

22. Roberts K, Dowell A, Nie JB. Attempting rigour and replicability in thematic analysis of qualitative research data; A case study of codebook development. BMC Med Res Methodol. 2019;19(1):1–8.

23. Boyatzis RE. Transforming qualitative information: Thematic analysis and code development. Transforming qualitative information: Thematic analysis and code development. Thousand Oaks, CA, US: Sage Publications, Inc; 1998. xvi, 184–xvi, 184.

24. Strauss A, Corbin J. Grounded Theory Methodology: AN Overview. In: Handbook of qualitative research. 1998.

25. Russo JA, Miller E, Gold MA. Myths and Misconceptions About Long-Acting Reversible Contraception (LARC). J Adolesc Heal. 2013 Apr;52(4):S14–21.

26. Ismail H, Mansour D, Singh M. Migration of Implanon®. J Fam Plan Reprod Heal

Care. 20061;32(3):157–9. Available from: https://jfprhc.bmj.com/lookup/doi/10.1783/147118906777888413

27. Garrett CC, Keogh LA, Kavanagh A, Tomnay J, Hocking JS. Understanding the low uptake of long-acting reversible contraception by young women in Australia: a qualitative study. BMC Womens Health. 2015;15(1):72.

28. Staveteig S, Mallick L, Winter R. Uptake and discontinuation of long-acting reversible contraceptives (LARCs) in low-income countries. DHS Analytical Studies No. 54 . Rockville, Maryland, USA : ICF International ; 2015. Available from: http://dhsprogram.com/pubs/pdf/AS54/AS54.pdf

29. Montgomery ET, Atujuna M, Krogstad E, Hartmann M, Ndwayana S, O'Rourke S, et al. The Invisible Product: Preferences for Sustained-Release, Long-Acting Pre-exposure Prophylaxis to HIV Among South African Youth. JAIDS J Acquir Immune Defic Syndr. 2019 Apr 15;80(5):542–50.

30. Kerrigan D, Mantsios A, Gorgolas M, Montes M-L, Pulido F, Brinson C, et al. Experiences with long acting injectable ART: A qualitative study among PLHIV participating in a Phase II study of cabotegravir + rilpivirine (LATTE-2) in the United States and Spain. Graham SM, editor. PLoS One. 2018 Jan 5;13(1):e0190487.

31. Blackstone SR, Nwaozuru U, Iwelunmor J. Factors Influencing Contraceptive Use in Sub-Saharan Africa: A Systematic Review. Int Q Community Health Educ. 2017 Jan 5;37(2):79–91.

32. Kriel Y, Milford C, Cordero J, Suleman F, Beksinska M, Steyn P, et al. Male partner influence on family planning and contraceptive use: perspectives from community members and healthcare providers in KwaZulu-Natal, South Africa. Reprod Health. 2019 Dec 25;16(1):89.

33. Nanda K, Callahan R, Dorflinger L. Addressing Gaps in the Contraceptive Method Mix: Methods in Development. Women's Heal. 2015 Nov;11(6):729–35.

34. Mwaisaka J, Gonsalves L, Thiongo M, Waithaka M, Sidha H, Agwanda A, et al. Exploring contraception myths and misconceptions among young men and women in Kwale County, Kenya. BMC Public Health. 2020;20(1):1694.

35. Bukenya D, Mayanja BN, Nakamanya S, Muhumuza R, Seeley J. What causes non-

adherence among some individuals on long term antiretroviral therapy? Experiences of individuals with poor viral suppression in Uganda. AIDS Res Ther. 2019;16(1):2.

36. Ontiri S, Ndirangu G, Kabue M, Biesma R, Stekelenburg J, Ouma C. Long-Acting Reversible Contraception Uptake and Associated Factors among Women of Reproductive Age in Rural Kenya. Int J Environ Res Public Health. 2019 May 1;16(9):1543.

37. Beesham I, Smit J, Bosman S, Milford C, Panday M, Beksinska ME. Women's contraceptive choice following the use of Implanon NXT: Findings from a study in Durban, South Africa. Afr J Reprod Health. 2021;25(1):41–8.

Ethics approval and consent to participate: The protocol, questionnaires, informed consent forms and study related materials were reviewed and approved by the University of KwaZulu-Natal Biomedical Research Ethics Committee (BE477/19) and the South African National Department of Health Ethics Committee (KZ_201910_017). All participants provided written informed consent prior to being enrolled.

Consent to publish: not applicable

Availability of data and materials: The data that support the findings of this study are available from CAPRISA but restrictions apply to the availability of these data, which were used under license for the current study, and so are not publicly available. Data are however available from the authors upon reasonable request and with permission of CAPRISA (data request form available at https://www.caprisa.org/).

Competing interests None.

Funding: Funding for the study was provided by South African Medical Research Council Special initiative grant (00251), and partial support from the Department of Science and Innovation-National Research Foundation Centre of Excellence in HIV Prevention.

Authors' Contributions: Contributors HH and MU conceptualised and designed the current analysis. HH completed the primary draft paper and completed all additional drafts after feedback from additional authors. HH, MU, GM, and MM were involved in data collection. All authors contributed to data analysis and interpretation. All authors contributed to either preparation or edits of the final manuscript and approved revisions.
